# Impferinnerungen in Deutschland: Bestandsaufnahme und Ideen für morgen am Beispiel der HPV-Impfung: Ergebnisbericht aus dem Projekt „InveSt HPV“

**DOI:** 10.1007/s00103-025-04030-8

**Published:** 2025-03-17

**Authors:** Anja Takla, Elisa Wulkotte, Yvonne Bichel, Johannes Lachmann, Angelika Trübswetter, Julia Wilhelm, Annabel Zettel, Nora Schmid-Küpke

**Affiliations:** 1 Fachgebiet Impfprävention, STIKO, Abteilung für Infektionsepidemiologie, Robert Koch-Institut, Berlin, Deutschland; 2 YOUSE GmbH, Berlin, Deutschland; 3 Robert Koch-Institut, Seestr. 10, 13353 Berlin, Deutschland

**Keywords:** Recall, HPV-Impfung, Evidenz, Akzeptanz, Equity, Recall, HPV vaccination, Evidence, Acceptance, Equity

## Abstract

Impfungen finden in Deutschland fast ausschließlich in Arztpraxen statt. Somit stellt jeder Praxis- bzw. Arztkontakt eine wichtige Chance für die Durchführung einer empfohlenen Impfung dar. Die Wirksamkeit von Einladungs- und Erinnerungssystemen für die Wahrnehmung von empfohlenen Impfungen wurde bereits durch Studien belegt, trotzdem werden solche Systeme in Deutschland – im Gegensatz zu vielen anderen Ländern – nicht flächendeckend genutzt. Die „Interventionsstudie zur Steigerung der HPV-Impfquoten in Deutschland“ (InveSt HPV) widmet sich daher u. a. der Frage, welche Hürden für den Einsatz bzw. die Verbreitung von Einladungs- und Impferinnerungssystemen bestehen. Dafür wurden 2 bundesweite quantitative Befragungen von (i) 345 niedergelassenen, kinderärztlich tätigen Ärzt:innen und (ii) 1805 Eltern mit Kindern im Alter von 9 bis 14 Jahren sowie eine Bestandsaufnahme bei 46 gesetzlichen Krankenkassen mit etwa 51 Mio. Versicherten durchgeführt. Die Befragungsergebnisse sowie weitere, durch das Projektteam zusammengetragene Evidenz waren Grundlage für einen Workshop mit impfrelevanten Akteur:innen aus der Gesundheitsversorgung. Ziel des Workshops war es, gemeinsam an Konzepten für ein zukünftiges Einladungs- und Impferinnerungssystem in Deutschland am konkreten Beispiel der Impfung gegen HPV (humane Papillomaviren) zu arbeiten. In dem vorliegenden Bericht werden Kernergebnisse der durchgeführten Befragungen und ausgewählte weitere, vom Projektteam für den Workshop zusammengetragene Evidenz vorgestellt. Schließlich werden der Workshop und die von den Teilnehmenden erarbeiteten zentralen Elemente für ein HPV-bezogenes Einladungs- und Impferinnerungssystem 2.0 beschrieben.

## Abstract (en)

In Germany, vaccinations take place almost exclusively in physicians’ private practices. This means that every contact with a practice or physician is an important opportunity to receive a recommended vaccination. The effectiveness of invitation and reminder systems for the implementation of recommended vaccinations has already been proven by studies, but in contrast to many other countries, such systems are not used across the board in Germany. The “Intervention Study to Increase HPV Vaccination Coverage in Germany” (InveSt HPV) therefore addresses, among other things, the question of what barriers exist to the use and dissemination of invitation and vaccination reminder systems. To this end, two nationwide quantitative surveys of (i) 345 pediatricians in private practices and (ii) 1805 parents with children aged 9 to 14 as well as a survey of 46 statutory health insurances with around 51 million insured persons were conducted. The survey results and other evidence compiled by the project team formed the basis for a workshop with vaccination-relevant stakeholders from the healthcare sector. The aim of the workshop was to work together on concepts for a future invitation and vaccination reminder system in Germany using the specific example of the HPV (Human Papillomavirus) vaccination. This report presents the core results of the surveys conducted and selected additional evidence compiled by the project team for the workshop. Finally, the workshop and the key elements developed by the participants for an HPV-related invitation and vaccination reminder system 2.0 are described.

## Einleitung

Impfungen gehören zu den wichtigsten und effektivsten gesundheitlichen Präventionsmaßnahmen. Empfehlungen für Impfungen werden in Deutschland von der Ständigen Impfkommission (STIKO) ausgesprochen [1]. Das Impfangebot und die Durchführung erfolgen in Deutschland hauptsächlich in Arztpraxen [2] im Rahmen eines opportunistischen Impfsystems [3]. Ein opportunistisches Impfsystem zeichnet sich dadurch aus, dass entweder medizinisches Personal bei passender Gelegenheit ein Impfangebot macht oder die zu impfende Person die Impfung in der Praxis aktiv nachfragt. Eine Gelegenheit für Impfangebote sind z. B. Gesundheits- bzw. Check-up-Untersuchungen [4]. In Deutschland sind vom Säuglings- bis zum Vorschulalter mehr als 9 Gesundheitsuntersuchungen in der kinderärztlichen oder hausärztlichen Versorgung vorgesehen. Ab Schuleintritt bis zum 18. Geburtstag sind es noch 3 Untersuchungen, von denen nur eine von allen Krankenkassen als Teil des Leistungskataloges bezahlt werden muss. Im Erwachsenenalter können betriebsmedizinische Untersuchungen sowie verschiedene, von den Krankenkassen als Regelversorgungsleistung übernommene Check-up-Untersuchungen in der Arztpraxis zur Impfbuchkontrolle und für mögliche Impfangebote genutzt werden [5].

Eine von der STIKO im Kindes- bzw. Jugendalter empfohlene Impfung ist die Impfung gegen humane Papillomaviren (HPV; [6]). Jährlich erkranken in Deutschland fast 8000 Menschen an HPV-bedingten Tumoren, die neben der häufigsten Lokalisation am Gebärmutterhals auch bei beiden Geschlechtern im Mund-Rachen-Raum und Anogenitalbereich auftreten können [7]. Die HPV-Impfempfehlung der STIKO gilt standardmäßig für alle Kinder und Jugendlichen von 9 bis 14 Jahren, eine Nachholimpfung ist bis zum 18. Geburtstag möglich. Als Gesundheitsuntersuchungen fallen in diesen Zeitraum die von allen Kassen als Regelversorgungsleistung zu übernehmende J1-Untersuchung (12–14 Jahre) und die freiwillig von einigen Krankenkassen übernommene U11 (9–10 Jahre; [4]). Obwohl die HPV-Impfung sehr wirksam vor HPV-bedingten Tumoren schützt, lagen die Impfquoten in Deutschland für eine vollständige HPV-Impfserie im Jahr 2023 bei den 15-jährigen Mädchen lediglich bei 55 % bzw. bei den gleichaltrigen Jungen bei 34 % [8]. Im europäischen Ländervergleich bewegte sich Deutschland damit im unteren Drittel [9]. Aufgrund des hohen Präventionspotenzials besteht sowohl vonseiten der Weltgesundheitsorganisation (WHO) als auch von der Kommission der Europäischen Union (EU) das Ziel, bis zum Jahr 2030 bei 15-jährigen Mädchen eine HPV-Impfquote von mindestens 90 % zu erreichen [10–12] und die Impfquote bei den gleichaltrigen Jungen deutlich zu steigern [11].

Im Gegensatz zu Deutschland nutzt die überwiegende Mehrheit der Länder in Europa strukturierte Impfsysteme [3]. In strukturierten Impfsystemen wird systematisch allen Personen innerhalb der Zielgruppe aktiv ein Impfangebot gemacht. Häufig beinhaltet ein strukturiertes Impfsystem auch Impferinnerungen der Zielgruppe [3]. Verschiedene Studien zeigen, dass Erinnerungssysteme einen positiven Effekt auf Impfquoten haben [13–15]. In Deutschland werden solche Systeme jedoch bisher nicht flächendeckend genutzt.

Daher beschäftigte sich die „Interventionsstudie zur Steigerung der HPV-Impfquoten in Deutschland“ (InveSt HPV) in einem von 2 Projektmodulen mit potenziellen Hürden für den Einsatz bzw. die Verbreitung von Einladungs- und Impferinnerungssystemen. Im Rahmen des Projektmoduls wurden 2 bundesweite quantitative Befragungen von (i) niedergelassenen, kinderärztlich tätigen Ärzt:innen sowie (ii) Eltern mit Kindern im Alter von 9 bis 14 Jahren durchgeführt. Ergänzt wurden die bundesweiten Befragungen durch eine Bestandsaufnahme bei gesetzlichen Krankenkassen. Durch die Befragungen soll die Nutzung von Impferinnerungssystemen aus verschiedenen Perspektiven untersucht werden: die Eltern als Empfänger:innen und die Pädiater:innen sowie Krankenkassen als potenzielle Absender:innen der Impferinnerungen. Schließlich wurde ein Workshop mit impfrelevanten Akteur:innen aus der Gesundheitsversorgung durchgeführt. Auf Basis der im Projektmodul zusammengetragenen Evidenz wurde gemeinsam am konkreten Beispiel der HPV-Impfung an möglichen Konzepten für ein zukünftiges Einladungs- und Impferinnerungssystem in Deutschland gearbeitet.

Im vorliegenden Bericht werden Kernergebnisse der Befragungen bei Pädiater:innen und Eltern sowie der Bestandsaufnahme bei den gesetzlichen Krankenkassen berichtet. Weitere, für den Workshop zusammengetragene Evidenz wird auszugsweise vorgestellt. Zum Schluss werden die im Workshop erarbeiteten Kernelelemente für ein Einladungs- und Impferinnerungssystem 2.0 in Deutschland in Kurzform dargelegt.

## Befragungen

Von August bis November 2023 beteiligten sich 345 von 6635 im Berufsverband der Kinder- und Jugendärzt*innen (BVKJ) organisierten niedergelassenen Pädiater:innen an dem Online-Survey. Im gleichen Zeitraum wurden auch 1805 Eltern mit mindestens einem 9‑ bis 14-jährigen Kind aus ganz Deutschland mittels Online-Survey befragt. Die Eltern wurden unter Berücksichtigung von Quoten nach Bildungsstand [16] und nach Geschlecht des Kindes (50 % Mädchen, 50 % Jungen) rekrutiert. Die Befragung der gesetzlichen Krankenkassen erfolgte von Oktober bis November 2023 (vor dem Inkrafttreten des Gesundheitsdatennutzungsgesetzes (GDNG) im März 2024 [17]) mittels eines standardisierten elektronischen Fragebogens in Form eines ausfüllbaren PDF-Dokuments. 46 Krankenkassen (von z. Zt. 95 [18]), die insgesamt etwa 51 Mio. Versicherte abdecken, nahmen an der Befragung teil. Nachfolgend finden sich die wichtigsten Ergebnisse der Befragungen. Methodik, Beschreibung der Studienpopulationen und weitere Ergebnisse finden sich im ausführlichen Projektbericht [19].

### Pädiater:innen

Ziel der Befragung der Pädiater:innen war es, deren Nutzung von Erinnerungssystemen für die HPV-Impfung sowie mögliche Hürden und Anreize dafür zu untersuchen. Die Kontrolle des Impfstatus erfolgte am häufigsten durch die Pädiater:innen selbst anhand der Patientenakte (27 % von *N* = 1002 Nennungen) oder des vorgelegten Impfausweises (21 %). Eine Kontrolle mittels Praxisverwaltungssoftware (PVS) beim Praxisbesuch wurde vergleichsweise selten (10 %) genannt. Anlässe für die Impfstatuskontrolle durch Pädiater:innen waren vor allem U‑ oder J‑Untersuchung und allgemein ein Praxisbesuch (47 % bzw. 38 % von 616 Nennungen). Zur Erinnerung an die HPV-Impfung nutzten Pädiater:innen mit 68 % am häufigsten das persönliche Gespräch in der Praxis, 11 % nutzten Erinnerungszettel (z. B. als Notizzettel am Impfpass) und 9 % eine App. Fast 75 % der Praxen, die primär schriftlich an die HPV-Impfung erinnerten, boten dies nur in deutscher Sprache an. 35 % der Pädiater:innen gaben an, in der Praxis ein softwaregestütztes Erinnerungssystem (SGE) zu nutzen. Ein Zusammenhang zwischen der Nutzung eines SGE und dem Praxisstandort (Ost/West; städtisch/ländlich), der personellen Praxisausstattung oder dem Alter der/des Praxisinhabenden wurde nicht beobachtet. Als häufigste Gründe für eine Nichtnutzung wurden die Auslastung der Praxis, der finanzielle Aufwand und der Zeitaufwand für den Versand von Erinnerungen genannt.

Praxen, die Erinnerungssysteme nutzten, setzten diese für verschiedene Leistungen ein: Am häufigsten wurden Früherkennungs- und Vorsorgeuntersuchungen (24 % bzw. 23 % von 388 Nennungen), STIKO-Standardimpfungen sowie die HPV-Impfung (jeweils 21 %) genannt. Von den SGE-Nutzer:innen gaben 60 % an, dass die Kontaktaufnahme zur HPV-Impferinnerung mit Patient:innen nichtautomatisiert durch das SGE, sondern durch das Praxispersonal erfolgt. Knapp die Hälfte der SGE-Nutzer:innen (48 %) erinnerten ihre Patient:innen einmalig an die HPV-Impfung, 35 % bei nicht erfolgter Impfung mehrmalig und 18 % mehrmalig unabhängig von einer bereits erfolgten Impfung. Die primäre Verantwortung für die Erinnerung an die HPV-Impfung sahen 58 % der Pädiater:innen bei den pädiatrischen Praxen und 28 % bei der Krankenkasse^1^, gefolgt von Gesundheitsamt (5 %), Landesgesundheitsbehörde (4 %) und Schule (2 %).

### Eltern mit Kindern zwischen 9 und 14 Jahren

In der Befragung von Eltern wurden ihre Erfahrung mit Impferinnerungssystemen sowie ihre Wünsche an Erinnerungen zur HPV-Impfung erhoben. Unabhängig vom Geschlecht ihres Kindes wurden 47 % der befragten Elternteile schon einmal an die HPV-Impfung erinnert, dies geschah signifikant häufiger bei Eltern mit einem hohen sozioökonomischen Status (SES), in städtischen Regionen oder bei Privatversicherten. Kinder, deren Eltern an die HPV-Impfung erinnert wurden, waren signifikant häufiger gegen HPV geimpft (70 % mind. einmal geimpft) als ohne Erinnerung (44 %, *p* < 0,001).

**Abb. 1:**
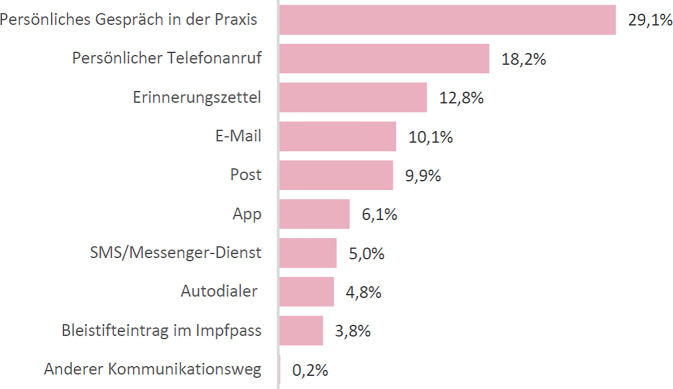
Von den Eltern angegebene Form der Erinnerung an die HPV-Impfung durch die Arztpraxis. Relativer Anteil an *N* = 1173 Nennungen. Quelle: eigene Abbildung

Am häufigsten wurden Eltern von der versorgenden Arztpraxis (76 % von 1260 Nennungen) oder der Krankenkasse (11 %) an die HPV-Impfung erinnert. Welche Kommunikationsform Arztpraxen und Krankenkassen dafür nutzten, ist in Abb. 1 und 2 dargestellt. Den Wunsch nach einer Impferinnerung äußerten 70 % der befragten Elternteile. Der Großteil bevorzugte eine Erinnerung, wenn ihr Kind im empfohlenen Impfalter ist (75 % vs. 21 % vor dem empfohlenen Alter). Der Erinnerungswunsch von Eltern bezog sich am häufigsten auf die versorgende Arztpraxis (57 % von 2719 Nennungen), gefolgt von der Krankenkasse (20 %). Am häufigsten wünschten sich die befragten Eltern die Erinnerung per Post (24 % von 3153 Nennungen), per E‑Mail (22 %) oder im persönlichen Gespräch in der Praxis (13 %). Eine personalisierte Erinnerung war 68 % der Eltern wichtig oder sehr wichtig. Auf die Frage, wer aus Elternsicht dafür verantwortlich sei, empfohlene Impfungen für ihr Kind im Blick zu behalten (Skala von 1 „gar nicht“ bis 5 „sehr stark“ verantwortlich), sahen Eltern die Verantwortung am häufigsten bei sich selbst (M = 4,2), gefolgt von der Arztpraxis (M = 3,8) und dem Gesundheitsamt (M = 3,2). Krankenkassen wurden in der Bewertung nicht abgefragt.

**Abb. 2:**
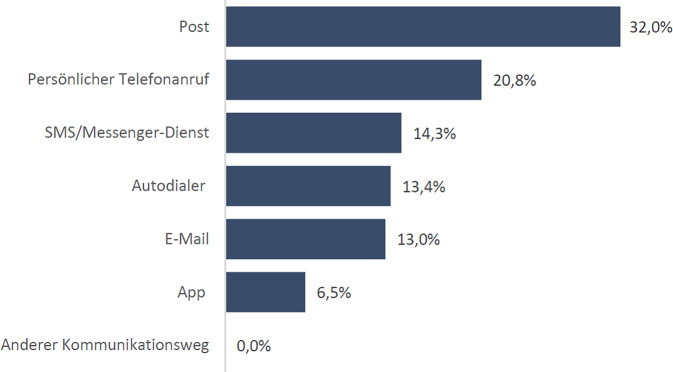
Von den Eltern angegebene Form der Erinnerung an die HPV-Impfung durch die Krankenkasse. Relativer Anteil an *N* = 231 Nennungen. Quelle: eigene Abbildung

### Krankenkassen

Die Befragung der gesetzlichen Krankenkassen beschränkte sich auf den Einsatz von Erinnerungssystemen für ihre Versicherten, Hürden oder Wünsche wurden nicht untersucht. Von den befragten gesetzlichen Krankenkassen nutzten 9 % keine Einladungs- oder Erinnerungssysteme. 91 % hatten diese Systeme für ihre Versicherten etabliert und wurden dazu näher befragt. Am häufigsten gaben die Vertreter:innen der Krankenkassen mit Einladungs- oder Erinnerungssystemen an, dass diese für Früherkennungsuntersuchungen genutzt werden (55 % der 71 Nennungen), gefolgt von der Nutzung für empfohlene STIKO-Standardimpfungen (18 %). Nur eine Krankenkasse evaluierte, ob die Einladung/Erinnerung zu einer Inanspruchnahme der Leistung geführt hat. 37 % gaben an, ihre Versicherten auch an die HPV-Impfung zu erinnern. Am häufigsten nutzten Krankenkassen dafür ihre Mitgliedszeitschrift/ihren Newsletter (32 % von 25 Nennungen), die krankenkasseneigene App (20 %), Post (20 %) oder E‑Mail (8 %). 43,5 % der befragten Krankenkassenvertreter:innen fänden es sinnvoll, den Impfstatus bei einer Einladung/Erinnerung zu berücksichtigen. Der Großteil (86 %) der Krankenkassen, die an die HPV-Impfung erinnerten, gab allerdings an, den Impfstatus ihrer Versicherten nicht ermitteln zu können.

### Zusammenfassung der Befragungsergebnisse

Kinder in Deutschland, deren Eltern an die HPV-Impfung erinnert wurden, waren signifikant häufiger gegen HPV geimpft. Am häufigsten erfolgte die HPV-Impferinnerung durch das persönliche Gespräch in der versorgenden Arztpraxis. Eltern dagegen wünschen sich eher schriftliche Erinnerungen. Die einer Erinnerung vorausgehende erforderliche Impfstatuskontrolle wurde von Pädiater:innen zumeist analog anhand der Akte oder des mitgebrachten Impfpasses durchgeführt. In den meisten Fällen war die Impfstatuskontrolle an eine U‑/J-Untersuchung gekoppelt. Softwaregestützte Erinnerungssysteme werden in Deutschland nur von einem Drittel der Pädiater:innen genutzt und sind damit bisher wenig verbreitet. Auch bei Pädiater:innen mit SGE erfolgte die Impferinnerung mehrheitlich und trotz hohen Zeitaufwands nicht durch automatisierte Prozesse, sondern durch das Praxispersonal. Auch wenn > 90 % der gesetzlichen Krankenkassen Einladungs‑/Erinnerungssysteme nutzten, entschied sich nur eine dieser Krankenkassen für eine Evaluierung, ob die Einladung/Erinnerung zu einer Inanspruchnahme der Leistung geführt hatte. Von den 40 % der Krankenkassen, die an HPV erinnern, gaben > 85 % an, den Impfstatus ihrer Versicherten nicht ermitteln können.

## Workshop

### Evidenzgrundlage

In Vorbereitung auf den Workshop wurde – neben den Befragungen – vom Projektteam weitere Evidenz zusammengetragen, um das Thema Einladungs- und Impferinnerungssysteme aus unterschiedlichen Blickwinkeln zu beleuchten. Den teilnehmenden Akteur:innen des Workshops sollten die Evidenzimpulse als Grundlage für eine informierte Diskussion dienen. Alle Impulse sind auf der Projektwebseite veröffentlicht [20]. Nachfolgend werden einige Inhalte der Impulse näher beschrieben, die aus Sicht der Autor:innen zentral sind, um über mögliche Konzepte für ein zukünftiges Einladungs- und Impferinnerungssystem in Deutschland zu diskutieren.

#### Etablierte Einladungs- und Erinnerungssysteme im Kinder- und Jugendalter in Deutschland

Auch wenn es in Deutschland kein strukturiertes Impfsystem gibt, findet sich in den meisten Bundesländern ein strukturiertes, gesetzlich geregeltes und verbindliches Einladungswesen für die Gesundheits- oder Früherkennungsuntersuchungen im Kindesalter (U1–U9; [21]). Unabhängig hiervon erfolgen Einladungen ggf. zusätzlich über die versorgende Arztpraxis oder jeweilige gesetzliche Krankenkasse bzw. private Krankenversicherung. Daten der letzten KiGGS-Welle 2 (2014–2017) zeigten, dass 97,2 % der Kinder die U3 bis U9 (ohne U7a) vollständig in Anspruch genommen hatten. Dieser Anteil lag unabhängig vom Geschlecht (Mädchen/Jungen), SES (niedrig/mittel/hoch) und Migrationshintergrund (ohne/einseitig/beidseitig) bei > 90 % [22]. Gleichzeitig fanden sich in den letzten Jahren konstant hohe Impfquoten (vollständige Impfserie) für die von der STIKO empfohlenen Standardimpfungen zum Zeitpunkt der Schuleingangsuntersuchung von > 80 % (z. B. Varizellen, Pneumokokken, Hepatitis B) bzw. > 90 % (Masern-Mumps-Röteln, Tetanus-Diphtherie-Pertussis-Polio-Hib, Meningokokken C; [23]). Die Schuleingangsuntersuchung im Alter von 5–6 Jahren [24] folgt zeitlich kurz nach der U9 (60.–64. Lebensmonat; [4]).

Im Gegensatz zu den U‑Untersuchungen besteht für die J1 im Alter von 12–14 Jahren kein vergleichbares Einladungswesen [21]. Letzte Analysen der Impfsurveillance aus Daten der kassenärztlichen Vereinigungen (KVen) zeigten J1-Teilnahmeraten zwischen 41 % und 46 % für die Geburtskohorten 2004–2007 (unveröffentlichte Daten, [25]). Die Teilnahmeraten waren vergleichbar für Jungen und Mädchen. Gleichzeitig zeigten 2 Studien aus Deutschland eine Assoziation von Praxiskontakt und HPV-Impfinanspruchnahme: Mädchen mit J1-Teilnahme im Alter von 12 Jahren hatten eine 7‑fach höhere Wahrscheinlichkeit, eine HPV-Impfung erhalten zu haben, als ohne J1-Teilnahme [26]. Mädchen mit U11-Teilnahme (9–10 Jahre) hatten eine deutlich höhere Chance, eine HPV-Impfung bekommen zu haben, als diejenigen, die die U11 nicht in Anspruch genommen hatten [27]. Die U11 ist aktuell kein Teil des Leistungskataloges der gesetzlichen Krankenkassen, jedoch prüft der Gemeinsame Bundesausschuss (G-BA) derzeit die Einführung einer zusätzlichen Früherkennungsuntersuchung für 9‑ bis 10-Jährige („neue U10“; [28]).

#### Impfen digital

Die Umsetzung von Impfempfehlungen kann durch verschiedene digitale Lösungen unterstützt werden, die gleichzeitig auch Impferinnerungen ermöglichen. Nachfolgend werden 2 digitale Lösungsansätze, die elektronische Patientenakte (ePA) und die Praxis-App „Meine pädiatrische Praxis“, exemplarisch vorgestellt, weitere Ansätze können z. B. das elektronische Impfmanagement in der Praxis sein [29].

Die *elektronische Patientenakte (ePA)* ist ein digitaler Speicherplatz für medizinische Unterlagen, den die Versicherten mithilfe von Apps ihrer gesetzlichen Krankenkasse einsehen und verwalten können. Die Apps haben zum Zeitpunkt, als das Manuskript verfasst wurde, identische Grundfunktionen, aber unterschiedliche Zusatzfunktionen. Initial enthält die ePA Medikationspläne, Arztbriefe, stationäre Entlassungsbriefe und Befundberichte in Form von medizinischen Informationsobjekten (MIO; [30]). Die ePA soll schrittweise erweitert werden, sodass perspektivisch auch Daten zu Impfungen im Rahmen eines elektronischen Impfpasses (eImpfpass) digital verfügbar gemacht werden [31]. Um die ePA nutzen zu können, ist von ärztlicher Seite eine Anbindung an die Telematikinfrastruktur notwendig, jedoch berichten etwa 2/3 von wöchentlichen oder täglichen Problemen mit ebendieser [32]. Vonseiten der Patient:innen werden eine „GesundheitsID“ und ein Zugang zu digitaler Infrastruktur benötigt. Seit dem 15.01.2025 ist die „ePA für alle“ nach Opt-out-Konzept vorgesehen. Versicherte erhalten automatisch eine ePA von ihrer gesetzlichen Krankenkasse und in der Gesundheitsversorgung kann darauf ohne erneute Freigabe der Versicherten zugegriffen werden – außer sie widersprechen aktiv. Der Bund hat sich als Ziel gesetzt, dass 80 % der gesetzlich Versicherten die ePA nutzen [33]. Umsetzungszeitplan und in Teilen auch die konkrete Ausgestaltung, wie z. B. eine mögliche Erinnerungsfunktion, sind jedoch aktuell noch ungeklärt.

Die *Praxis-App Meine pädiatrische Praxis* (vormals „Mein Kinder- und Jugendarzt“) wurde von einem privaten Anbieter in Zusammenarbeit mit dem BVKJ entwickelt und wird mittlerweile von > 1200 Praxen bzw. etwa 40 % aller Kinder- und Jugendärzt:innen genutzt (persönliche Kommunikation, BVKJ). Praxen können sich gegen Gebühr für die App-Nutzung registrieren, müssen die App aber selbst aktiv bespielen. Die App bietet verschiedene Funktionen: Unter anderem können Eltern an Termine, Vorsorgeuntersuchungen und Impfungen erinnert werden. Für Impfungen können sogenannte Impfsteckbriefe mit Erinnerung versandt werden. Eltern können die App nur nutzen, wenn die versorgende Praxis registriert ist, und müssen die Daten zum Kind selbst eingeben. Impferinnerungen finden auf Grundlage des von den Eltern eingegebenen Alters statt. Personalisierte Impferinnerungen (d. h. in Abhängigkeit vom tatsächlichen Impfstatus) können (bisher) nicht versandt werden, da es derzeit keine Schnittstelle zur Praxisverwaltungssoftware gibt [34].

### Einladungssystem für alle: Wer mit welchen Daten wen erreichen kann

**Abb. 3:**
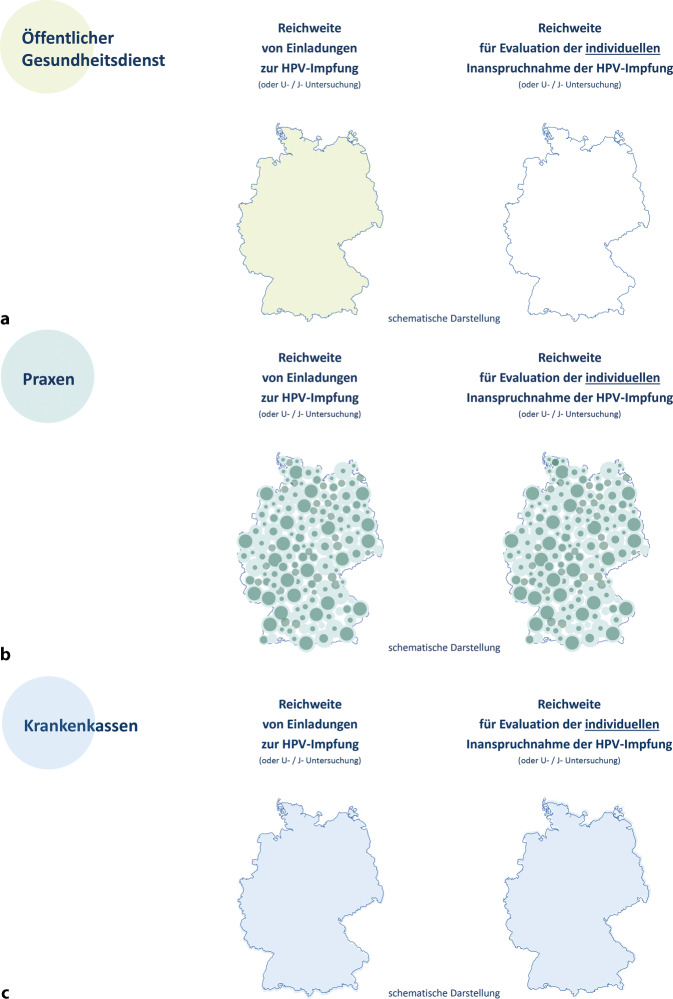
Reichweite von Einladungen zur Impfung gegen humane Papillomaviren (HPV) und einer möglichen Evaluation der individuellen Inanspruchnahme der HPV-Impfung. **a** Öffentlicher Gesundheitsdienst: Antizipierte Reichweite ist in lindgrün dargestellt. **b** Ärztliche Praxen: Antizipierte Reichweite der einzelnen Praxen umfasst den bestehenden Patientenstamm mit aktiver Arzt-Patient-Beziehung (*dunkelgrün*) und ggf. mit inaktiver Arzt-Patient-Beziehung (*hellgrün*). **c** Krankenkassen (umfasst gesetzliche Krankenkassen und private Krankenversicherungen): Antizipierte Reichweite ist in *hellblau* dargestellt. Quelle: eigene Abbildung

Für das Konzept eines strukturierten Einladungs- und Impferinnerungssystems sind 2 Fragen zentral: Wer kann mit welchen Daten wen erreichen? Mit welchen Daten kann eine Inanspruchnahme der Leistung evaluiert werden? Diese Fragen sind v. a. im Hinblick auf Zugangsgerechtigkeit (Equity) zentral [35]. Die Abb. 3a–c zeigen die mögliche Reichweite von Einladungen zur HPV-Impfung und einer Evaluation der Inanspruchnahme für die 3 wichtigsten Akteure Öffentlicher Gesundheitsdienst (ÖGD), Praxen und Krankenkassen (bzw. -versicherungen; [36]).

#### Öffentlicher Gesundheitsdienst.

Für eine postalische Kontaktaufnahme stehen dem ÖGD Daten aus den Einwohnermeldeämtern zur Verfügung (Abb. 3a). Diese könnten auch Daten zur Nationalität der zu kontaktierenden Person enthalten, die einen wichtigen Hinweis auf (weitere) gesprochene Sprachen und einen möglichen Bedarf für mehrsprachiges Einladungs- und Aufklärungsmaterial geben könnten. Auf Daten, die eine Evaluation der Inanspruchnahme ermöglichen würden, hat der ÖGD keinen Zugriff.

#### Ärztliche Praxen.

Einladungen zur HPV-Impfung durch ärztliche Praxen (Abb. 3b) setzen aktuelle Kontaktdaten und Einwilligungserklärungen voraus, die am ehesten bei einer aktiven Arzt-Patient-Beziehung vorhanden sind (dunkelgrüne Kreise). Gegebenenfalls lassen sich auch Patient:innen mit inaktiver Arzt-Patient-Beziehung oder ehemalige Patient:innen mit noch aktuellen Kontaktdaten erreichen (hellgrüne Kreise). Es kann davon ausgegangen werden, dass Praxen den Bedarf von mehrsprachigem Einladungs- und Aufklärungsmaterial einschätzen können. Eine Evaluation der individuellen Impfinanspruchnahme kann für alle Patient:innen mit einer aktiven Arzt-Patient-Beziehung erfolgen. Es gilt aber zu beachten, dass nicht der gesamten Bevölkerung ein pädiatrisches Angebot zur Verfügung steht: In einer Umfrage des Deutschen Kinderhilfswerks von 2018 gaben 34 % der Eltern an, keine ausreichende pädiatrische Versorgung in ihrer Nähe zu haben [37]. In der KiGGS-Welle 2 (2014-2017) gaben 12 % der Kinder und Jugendlichen an, dass sie im letzten Jahr keine ambulante pädiatrische Versorgung in Anspruch genommen hatten [22]; in der Online-Befragung der Eltern im Rahmen von InveSt HPV betraf dies 7 % der 9‑ bis 14-Jährigen [19].

#### Gesetzliche Krankenkassen und private Krankenversicherungen.

Mit Stand 2024 gab es in Deutschland etwa 130 Krankenkassen und -versicherungen [18, 38]. Entsprechend der Krankenversicherungspflicht für alle Bürger:innen mit Wohnsitz in Deutschland [39] sind laut Mikrozensusdaten 99,9 % der Bevölkerung in Deutschland krankenversichert [40]. Die potenzielle Reichweite von Krankenkassen und -versicherungen wird in Abb. 3c veranschaulicht. Krankenkassen und -versicherungen verfügen über aktuelle Daten für nahezu die gesamte Zielgruppe: Ihnen liegen u. a. Daten zu Alter, Impfstatus und Nationalität der versicherten Person vor, sodass auch der Bedarf für mehrsprachiges Einladungs- und Aufklärungsmaterial abgeschätzt werden könnte. Für die Evaluation der individuellen Inanspruchnahme von Leistungen können Abrechnungsdaten für ärztliche Leistungen (mit zeitlichem Verzug) genutzt werden.

Es muss jedoch darauf hingewiesen werden, dass von einigen Krankenkassen starke Bedenken geäußert wurden, ob Evaluationen der Inanspruchnahme von Leistungen für personalisierte Erinnerungen juristisch zulässig sind. Darauf weisen auch die hier vorgestellten Befragungsergebnisse bei den Krankenkassen hin, in der ein Großteil der Krankenkassenvertreter:innen angab, den Impfstatus nicht ermitteln zu können. Obwohl im März 2024 das GDNG [17] in Kraft treten sollte, das durch Einführung des § 25b SGB V den Kranken- und Pflegekassen die Möglichkeit zur datengestützten Auswertung zur Erkennung individueller Gesundheitsrisiken einräumt [41, 42], wurden weiterhin Bedenken geäußert. Aus diesem Grund gab es in dem anstehenden Workshop einen eigenen Evidenzimpuls zur juristischen Beurteilung der rechtlichen Grundlagen für personalisierte Impferinnerungen von gesetzlichen Krankenkassen und privaten Krankenversicherungen [43].

### Durchführung des Workshops

**Abb. 4:**
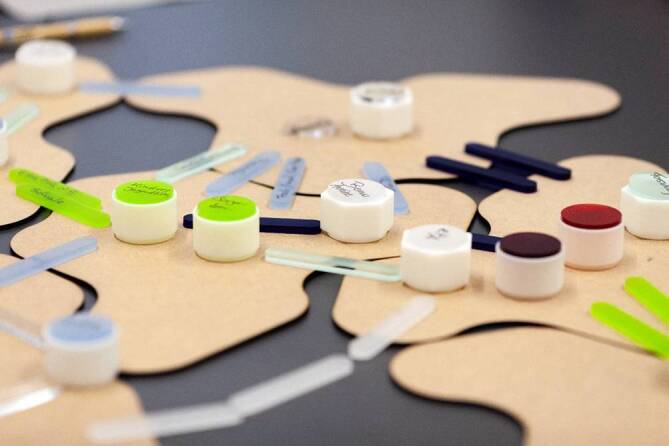
Unterschiedliche Workshop-Materialien, mit denen die in den Kleingruppen diskutierten Elemente für ein Einladungs- und Impferinnerungssystem in Deutschland 3‑dimensional sichtbar gemacht werden können; „InveSt HPV“-Workshop: „Einladungs- und Impferinnerungssysteme 2.0“, 12./13.04.2024, Berlin. Copyright: RKI/Zellentin

Am 12. und 13.04.2024 wurde in Berlin ein Workshop mit relevanten Akteur:innen aus der Gesundheitsversorgung durchgeführt. Ziel war es, Konzeptvorschläge für ein Einladungs- und Impferinnerungssystem in Deutschland zu erarbeiten. Am Workshop nahmen Vertreter:innen von gesetzlichen Krankenkassen (GKV) und privaten Krankenversicherungen (PKV), dem GKV-Spitzenverband, dem BVKJ, der Deutschen Gesellschaft für Allgemeinmedizin und Familienmedizin (DEGAM), dem Bundesministerium für Gesundheit (BMG), den Ländern, der Bundeszentrale für gesundheitliche Aufklärung (BZgA), der Nationalen Lenkungsgruppe Impfen (NaLI), dem Institut für Qualität und Wirtschaftlichkeit im Gesundheitswesen (IQWiG) und dem Leibniz-Institut für Präventionsforschung und Epidemiologie (BIPS) teil. Im Anschluss an kurze „Evidenzimpulse“ wurden die Teilnehmenden in Kleingruppen aufgeteilt. Gemeinsam wurde über die aus Sicht der Teilnehmenden wichtigen Elemente für ein Konzept diskutiert. Die Kleingruppen nutzten unterschiedliche Materialien, um das Konzept mit den dazugehörigen Elementen 3‑dimensional sichtbar zu machen (Abb. 4). Dabei brachten die Akteur:innen ihre unterschiedlichen Perspektiven und Erfahrungen ein, was v. a. für die Diskussion der Praktikabilität der Konzepte zentral war. Die von den Gruppen entwickelten Konzeptbausteine wurden zum Schluss im Plenum vorgestellt und diskutiert.

### Mögliche Elemente für Einladungs- und Impferinnerungssysteme in Deutschland

Als zentrale Voraussetzung hoben die Teilnehmenden eine kooperative und effiziente Zusammenarbeit der Akteur:innen hervor, um Synergien zu nutzen und mögliche Doppelstrukturen abzubauen oder zu vermeiden. Über die verschiedenen Gruppen hinweg gab es bestimmte Kernelemente, die von allen als wichtig und zielführend für die Erinnerung an die HPV-Impfung erachtet wurden.

Das zentralste Element war die Erweiterung des Vorsorgeverständnisses bis zum 18. Geburtstag. Konkret wurde die Etablierung der U11 im Alter von 9–10 Jahren (bzw. der aktuell vom G‑BA geprüften „neuen U10“ im Alter von 9–10 Jahren) und ggf. der J2 als weitere gesetzliche Früherkennungsuntersuchung (und damit Aufnahme in den Leistungskatalog der gesetzlichen Krankenkassen) diskutiert. Diese Gesundheits- oder Früherkennungsuntersuchungen wurden als wichtige strukturierte Praxiskontakte angesehen, um die Zielgruppe zur HPV-Impfung zu beraten bzw. die Impfung zeitgerecht durchzuführen. Die Erweiterung des aktuell bis zur U9 etablierten Systems bietet aufgrund der verlässlich hohen Teilnahmeraten [22] die Chance, alle Eltern und Kinder in die Praxis einzuladen und damit eine Gelegenheit zur HPV-Impfung zu schaffen. Um Eltern die Relevanz der U11 (bzw. der „neuen U10“) und der J1 zu verdeutlichen, sollte das mit der U9 endende „Gelbe Heft“ um diese Untersuchungen erweitert und die Verlängerung des Einladungs- und Rückmeldesystems der Länder bis zur U11 (bzw. zur „neuen U10“) und zur J1 initiiert werden.

Weitestgehend einig waren sich die Teilnehmenden auch zum „eImpfpass“ als Teil der „ePA für alle“. Um diese für alle Beteiligten (Patient:innen/Versicherte, Praxispersonal, Krankenkassen) niederschwellig nutzbar zu gestalten, sind Schnittstellen zu anderen Softwaresystemen, wie dem Praxisverwaltungssystem, notwendig. Der eImpfpass kann so ausgestaltet werden, dass Nutzer:innen Erinnerungen an die HPV-Impfung sowie andere Impfungen unter Berücksichtigung von Alter und Impfstatus erhalten. Um die ePA als Grundlage für ein Einladungs- und Impferinnerungssystem nutzen zu können, sollte sich schnellstmöglich auf einen Umsetzungszeitplan geeinigt werden. Für die konkrete Ausgestaltung der Funktionen sollten relevante Akteure einbezogen und die Akzeptanz der Eltern für das System berücksichtigt werden. Wichtige Fragen für eine Impferinnerungsfunktion sind: Welche Informationen enthält eine Erinnerung (z. B. Termin, Aufklärungsmaterialien)? Wie können – im Sinne des Equity-Gedankens – Personen eingeladen und ggf. erinnert werden, die die ePA durch Opt-out nicht nutzen?

## Fazit

Verschiedene Studien haben bereits gezeigt, dass Erinnerungssysteme einen positiven Effekt auf Impfquoten haben. Auch die im Rahmen des Projektes durchgeführten Befragungen belegen für Deutschland einen Zusammenhang von Erinnerungssystemen und HPV-Impfstatus: Kinder, deren Eltern an die HPV-Impfung erinnert wurden, waren signifikant häufiger gegen HPV geimpft. Die Befragungsergebnisse machen jedoch auch deutlich, dass HPV-Impferinnerungen in Deutschland derzeit nicht systematisch erfolgen und zumeist an eine aktive Arzt-Patient-Beziehung mit Praxisbesuchen geknüpft sind. Kinder, die nicht oder nicht regelmäßig kinderärztlich versorgt werden, sind damit bisher meist von Impferinnerungen ausgeschlossen. Gesetzliche Krankenkassen gaben an, dass zwar Einladungs- und Erinnerungssysteme grundsätzlich genutzt werden, jedoch vorrangig für Früherkennungsuntersuchungen. Zum Zeitpunkt der Befragung (vor Inkrafttreten des GDNG) evaluierten Krankenkassen nicht, ob die Einladungen/Erinnerungen zu einer Inanspruchnahme der Leistung führten. Zudem gaben fast alle Krankenkassen mit HPV-Impferinnerung an, den HPV-Impfstatus ihrer Versicherten nicht ermitteln zu können – was personalisierte Impferinnerungen durch Krankenkassen *per se* ausschließen würde. Diese Einschätzung wurde während des Workshops trotz zwischenzeitlichen Inkrafttretens des GDNG von Krankenkassenvertreter:innen teilweise weiterhin vertreten. Um Krankenkassen und -versicherungen als Akteure für personalisierte Einladungs- und Impferinnerungssysteme berücksichtigen zu können, müssten ggf. noch bestehende offene rechtliche Unsicherheiten oder Vorbehalte gegenüber der Nutzung von Impfdaten für eine Erinnerung der Versicherten geklärt bzw. ausgeräumt werden.

Die Ergebnisse zeigen ebenfalls, dass bei allen Überlegungen über zukünftige Konzepte für Einladungs- und Impferinnerungssysteme zwingend berücksichtigt werden muss, welche Akteure alle Personen in der Zielgruppe sicher und unabhängig von Merkmalen, wie z. B. SES, Wohnort oder Versicherungsart, erreichen können. Nur so kann Zugangsgerechtigkeit (Equity) sichergestellt werden.

Ein pragmatischer Ansatz, der auch im Rahmen des Projekt-Workshops Konsens fand, könnte darin bestehen, ein Einladungs- und Impferinnerungssystem für das Kinder- und Jugendalter an das bereits etablierte und durch hohe Teilnahmeraten erfolgreiche System der Früherkennungsuntersuchungen bis zum Schuleintritt U3–U9 anzukoppeln. Dies beinhaltet die Aufnahme der U11 (bzw. der „neuen U10“) in den Leistungskatalog der Krankenkassen und die Erweiterung des strukturierten, gesetzlich geregelten und verbindlichen Einladungswesens in den Ländern um die U11 (bzw. „neue U10“) und die J1. Damit einhergehen sollte die Erweiterung des „Gelben Heftes“ um die 3 bis zum 18. Geburtstag vorhandenen Vorsorgeuntersuchungen U11 (bzw. „neue U10“), J1 und J2. So können verlässliche Praxiskontakte und zeitgerechte Impfangebote auch im Jugendalter geschaffen werden. Studien haben bereits gezeigt, dass die Inanspruchnahme der J1-Untersuchung mit einer höheren Wahrscheinlichkeit für die HPV-Impfung einhergeht.

Perspektivisch werden in den nächsten Jahren flächendeckende digitale Möglichkeiten, wie z. B. „ePA“ und „eImpfpass“, neue Möglichkeiten für Einladungs- und Impferinnerungssysteme bieten. Die konkrete Ausgestaltung sollte unter Einbeziehung der relevanten Akteure erfolgen und berücksichtigen, dass eine hohe Akzeptanz des digitalen Systems entscheidend für seine Nutzung und damit auch seine Wirkung ist. Dabei sollten auch Erfahrungen bestehender und funktionierender, wenn auch nicht flächendeckender Systeme berücksichtigt werden. Gerade Erwachsene und Senior:innen mit häufig niedrigen Impfquoten für STIKO-empfohlene Standard- und Indikationsimpfungen sind eine weitere wichtige Zielgruppe, die in einem nationalen Konzept für ein Einladungs- und Impferinnerungssystem speziell mitgedacht werden muss. Die Implementierung so eines strukturierten nationalen Systems ist eine vielversprechende Strategie, um nicht nur die Inanspruchnahme der HPV-Impfung, sondern aller STIKO-empfohlenen Impfungen zu fördern und gleichzeitig einen gerechten Zugang zu Impfangeboten für alle Personen in Deutschland zu gewährleisten.

## Footnotes

1 Der Terminus „Krankenkasse“ umfasste in der Pädiater:innen- und Elternbefragung sowohl gesetzliche Krankenkassen als auch private Krankenversicherungen.
